# *In Vivo* and *In Vitro* Impact of Carbohydrate Variation on Human Follicle-Stimulating Hormone Function

**DOI:** 10.3389/fendo.2018.00216

**Published:** 2018-05-09

**Authors:** George R. Bousfield, Jeffrey V. May, John S. Davis, James A. Dias, T. Rajendra Kumar

**Affiliations:** ^1^Department of Biological Sciences, Wichita State University, Wichita, KS, United States; ^2^Department of Obstetrics and Gynecology, University of Nebraska Medical Center, Omaha, NE, United States; ^3^Department of Biochemistry and Molecular Biology, University of Nebraska Medical Center, Omaha, NE, United States; ^4^Nebraska-Western Iowa Health Care System, Omaha, NE, United States; ^5^Department of Biomedical Sciences, School of Public Health, University at Albany, Albany, NY, United States; ^6^Department of Obstetrics and Gynecology, University of Colorado Anschutz Medical Campus, Aurora, CO, United States

**Keywords:** pituitary, N-glycosylation, follicle-stimulating hormone, bone, female Infertility

## Abstract

Human follicle-stimulating hormone (FSH) exhibits both macro- and microheterogeneity in its carbohydrate moieties. Macroheterogeneity results in three physiologically relevant FSHβ subunit variants, two that possess a single N-linked glycan at either one of the two βL1 loop glycosylation sites or one with both glycans. Microheterogeneity is characterized by 80 to over 100 unique oligosaccharide structures attached to each of the 3 to 4 occupied N-glycosylation sites. With respect to its receptor, partially glycosylated (hypo-glycosylated) FSH variants exhibit higher association rates, greater apparent affinity, and greater occupancy than fully glycosylated FSH. Higher receptor binding-activity is reflected by greater *in vitro* bioactivity and, in some cases, greater *in vivo* bioactivity. Partially glycosylated pituitary FSH shows an age-related decline in abundance that may be associated with decreased fertility. In this review, we describe an integrated approach involving genetic models, *in vitro* signaling studies, FSH biochemistry, relevance of physiological changes in FSH glycoform abundance, and characterize the impact of FSH macroheterogeneity on fertility and reproductive aging. We will also address the controversy with regard to claims of a direct action of FSH in mediating bone loss especially at the peri- and postmenopausal stages.

## Structural Attributes of Follicle-Stimulating Hormone (FSH) and its Subunits

Follicle-stimulating hormone is one of three gonadotropins in the human glycoprotein hormone family. This hormone family is part of the cystine knot growth factor superfamily, a large group of homo- and heterodimeric signaling molecules (1). FSH plays a central role in reproduction, particularly in females. In the ovary, FSH stimulates follicle development and estrogen synthesis. In the testis, FSH maintains Sertoli cell function, which supports spermatogenesis. Although currently controversial (2, 3), FSH has been claimed to play a direct role in osteoporosis by stimulating differentiation of osteoclasts, which are responsible for removing bone (4). The idea put forth is that in the postmenopausal period when FSH levels rise, activation of osteoclasts results in bone loss. Reports of non-gonadal actions of FSH have recently been summarized (5).

**Figure 1:**
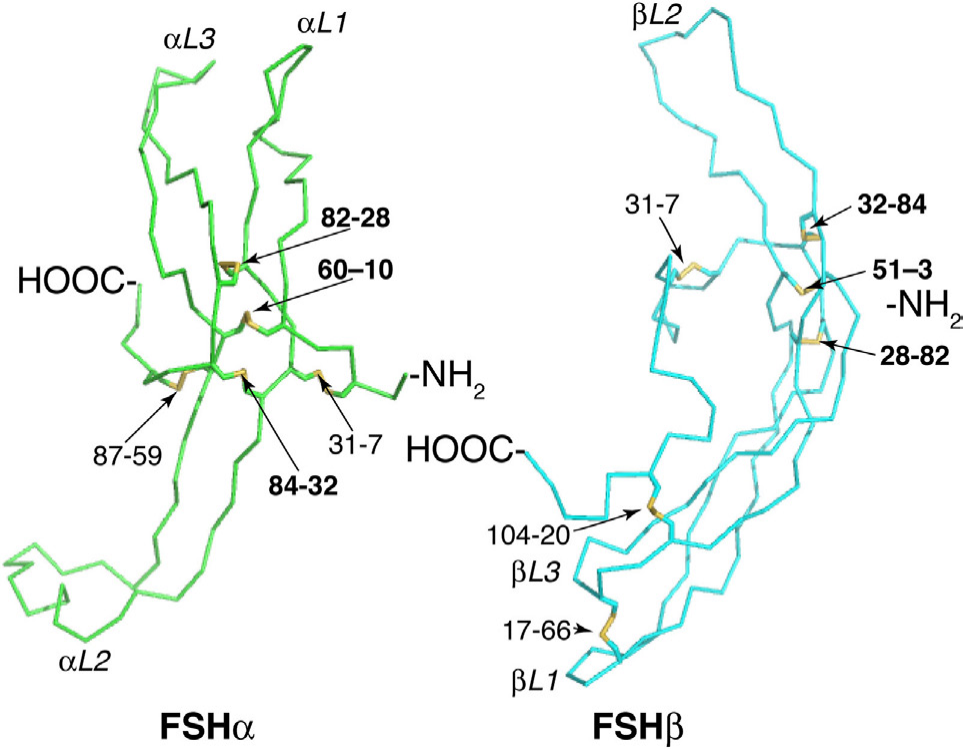
Follicle-stimulating hormone (FSH) subunit peptide moieties. Wire-frame models of FSH subunits extracted from pdb 1FL7 using MacPyMOL v1.8.2.3. FSHα backbone is green and FSHβ backbone is cyan. Disulfide bonds are indicated as yellow sticks. Cystine knot loops are designated by subunit (α or β) and number (1–3). Pairs of numbers refer to Cys residues involved in a disulfide bond. Bold numbers indicate Cys Knot disulfide bonds.

Follicle-stimulating hormone is composed of two dissimilar, cystine knot motif glycoprotein subunits: a common α-subunit and hormone-specific β-subunit (Figure 1) (6). The FSHα subunit amino-acid sequence and disulfide bond organization, including a cystine knot motif, are identical to those in the other glycoprotein hormones, luteinizing hormone (LH), thyroid-stimulating hormone (TSH), and chorionic gonadotropin (CG) (7). However, the N-glycan populations at both glycosylated residues, Asn^52^ and Asn^78^, differ from those of the other glycoprotein hormone α-subunits such that these otherwise identical subunits can be distinguished from each other and from free α-subunit by their oligosaccharide populations (8–10). The hormone-specific FSHβ subunit shares 34–40% sequence homology, six conserved disulfide bonds, cystine knot motif, and seatbelt loop with the other human glycoprotein hormone β-subunits (7, 11, 12). While there are two potential N-glycosylation sites in FSHβ, partially glycosylated variants exist that are missing either one of these oligosaccharides (13). These contribute to an unknown degree of charge variation in FSH preparations and result in the classic FSH isoforms (14, 15). The classic interpretation of FSH isoforms was based solely on the notion that variant patterns of negatively charged sialic acid or, to a much lesser extent, sulfate residues terminated oligosaccharide branches, which gave rise to differentially charged isoforms. The observation of hypo-glycosylation further refines our understanding of isoforms, in that net charge may vary, due to presence or absence of entire glycans.

## FSH Glycosylation Heterogeneity

Follicle-stimulating hormone glycosylation exhibits both macro- and microheterogeneity (Table 1). Macroheterogeneity herein refers to the presence or absence of glycosylation at any one potential glycosylation site. Examples of FSH macroheterogeneity involve the absence of either FSHβ Asn^7^ or Asn^24^ oligosaccharides in a population of fully processed and secreted FSH. Microheterogeneity herein refers to as many as 80 to over 100 unique oligosaccharide structures, which can be detected once released from each of the 3–4 glycan-occupied Asn residues in FSH.

**Table 1:** Macro- and microheterogeneity of hFSH preparations.

	Macroheterogeneity (% relative abundance)						
FSH preparation	Pituitary hFSH	Urinary hFSH	Pituitary hFSH^24^	Pituitary hFSH^21^	Pituitary hFSH^21/18^	Recombinant GH_3_ hFSH^24^	Recombinant GH_3_ hFSH^21^
FSH^24^	77	86	100	–	–	89	–
FSH^21^	23	14	–	100	60	11	54
FSH^18^	–^a^	–	–	–	40	–	46
FSH^15^	–	–	–	–	–	–	–

	**Types of oligosaccharides (% relative abundance)**						
**Oligosaccharide type**						**Recombinant GH_3_-hFSH^b^**	

Biantennary	38.2	37.2	47.1	51.2	28.6	55.5	
Triantennary(3)^c^	41.0	44.0	30.7	35.9	2.5	0	
Triantennary(6)^d^	0	0	0	0	0	29.7	
Tetra-antennary	15.0	14.8	10.6	6.0	0.01	0	
Neutral	0.3	2.2	9.9	4.5	74.2	12.3	
Sialylated	99.1	97.5	75.4	78.8	20.7	87.7	
Sulfated	6.5	4.2	39.3	35.0	9.6	0	
Sial/sulfat	5.9	3.9	24.0	18.3	4.5	0	
Core fucose	43.0	23.9	45.1	47.8	23.0	50.6	
Antenna-fucose	0.3	0	3.6	0.8	0.4	19.9	
Bisect GlcNAc	32.6	23.9	17.9	23.2	7.9	47.0	
GalNAc	2.8	1.7	20.3	13.8	14.1	10.5	

*Relative abundance determined by Western blot and mass spectrometry, respectively. Data are limited to those preparations for which both glycoform abundance and glycan microheterogeneity exist*.

*^a^– = not detected*.

*^b^ = glycoforms not separated*.

*^c^Triantennary(3) = third branch attached to Man(α1–3) branch*.

*^d^Triantennary(6) = third branch attached to Man(α1–6) branch*.

*Data derived from Ref. (16–18). FSH, follicle-stimulating hormone*.

**Figure 2:**
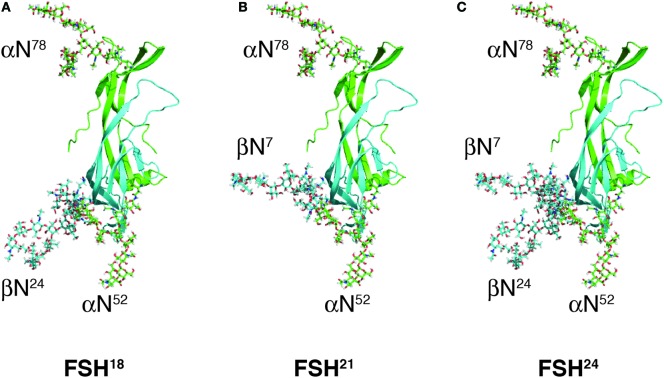
Follicle-stimulating hormone (FSH) glycoform models. Models of FSH heterodimers extracted from pdb 4AY9 decorated with the most abundant glycan observed at each N-glycosylation site by nano-ESI-ion mobility-MS (Bousfield, G. R. and Harvey, D. J., unpublished). Subunits are shown as cartoons rendered by MacPyMOL with subunits and their oligosaccharides colored as in Figure 1; FSHα green and FSHβ cyan. Oligosaccharides shown as sticks were created and attached to the FSH model using GLYCAM [Woods Group. (2005–2017) GLYCAM Web. Complex Carbohydrate Research Center, University of Georgia, Athens, GA, USA. (http://glycam.org)]. **(A)** FSH^18^, which lacks Asn^24^ glycan. **(B)** FSH^21^, which lacks Asn^7^ glycan. **(C)** FSH^24^, which possesses all four N-glycans.

Differences in electrophoretic mobility of FSH subunits, revealed by subunit-specific Western blots, provide a convenient means to distinguish four FSH variants resulting from macroheterogeneity. Fully glycosylated hFSHβ migrates as a 24-kDa band (hereinafter, 24k-FSHβ), desN^24^glycan-FSHβ migrates as a 21-kDa band (21k-FSHβ), and desN^7^glycan-FSHβ migrates as an 18-kDa band (18k-FSHβ). The FSH heterodimers that incorporate these β-subunit variants are designated, FSH^24^, FSH^21^, and FSH^18^, respectively (19), and are shown in Figure 2. Pituitary extracts also possess a non-glycosylated, 15-kDa FSHβ variant (20). However, the corresponding FSH^15^ does not appear to be physiologically relevant, because subunit association is extremely inefficient when both FSHβ glycans are missing, and little, if any, FSH heterodimer is secreted (21). FSH^24^ and FSH^21^ are detected in FSH derived from human pituitary extracts, as well as from urinary protein preparations (Table 1). When FSH is separated into fully- and hypo-glycosylated fractions, the latter often include FSH^18^, which can constitute as much as 40% of the hypo-glycosylated FSH preparation (13). As most hFSH^21^ preparations also possess hFSH^18^, and are not easily separated, it has become a convention to abbreviate the mixture of physiologically relevant hypo-glycosylated FSH preparations as hFSH^21/18^.

Follicle-stimulating hormone microheterogeneity results from a structurally heterogeneous population of oligosaccharides attached to each glycosylated Asn residue of the four glycosylation sequons in FSH. Microheterogeneity in this hormone has largely been evaluated at the whole hormone level in studies of pituitary and urinary FSH preparations (16, 22–25). Human pituitary FSH oligosaccharides are 85–98% complex-type, 88–99% are sialylated, 36–46% are biantennary, 30–49% are triantennary, 5–15% are tetra-antennary, while only 4–7% are sulfated (Table 1). The low extent of oligosaccharide sulfation appears to be a human-specific characteristic (no data exist for nonhuman primate FSH glycans), as FSH preparations from cattle, pigs, sheep, and horses possess higher levels of sulfated oligosaccharides, ranging from 13 to 58% (23, 26). Accordingly, a major factor in determining hFSH clearance rates is the extent of sialic acid termination at the non-reducing ends of oligosaccharide branches. As compared with naturally occurring hFSH preparations, recombinant hFSH preparation oligosaccharides exhibit a reduced degree of branching, consisting of largely (55%) biantennary glycans. However, the degree of sialylation in these preparations lags that of urinary hFSH to a lesser extent, because the most abundant urinary FSH triantennary and tetra-antennary glycans are one sialic acid residue short of a full complement (16, 25, 27).

As mentioned above, microheterogeneity contributes to charge variation in FSH, and this has been reported to alter FSH biological activity (14, 28, 29). Comparisons of microheterogeneity in early studies were challenged not only by the large number of oligosaccharide structures encountered, but also by the different analytical methods each group employed, as each of these exhibited bias toward or against specific families of oligosaccharides. We recently characterized microheterogeneity in three purified human pituitary FSH glycoform preparations, as well as highly purified pituitary, urinary, and recombinant hFSH preparations using nano-electrospray mass spectrometry (13, 16–18). Because over 33–109 structures were detected in each sample, comparing oligosaccharide populations derived from different FSH preparations proved challenging.

**Figure 3:**
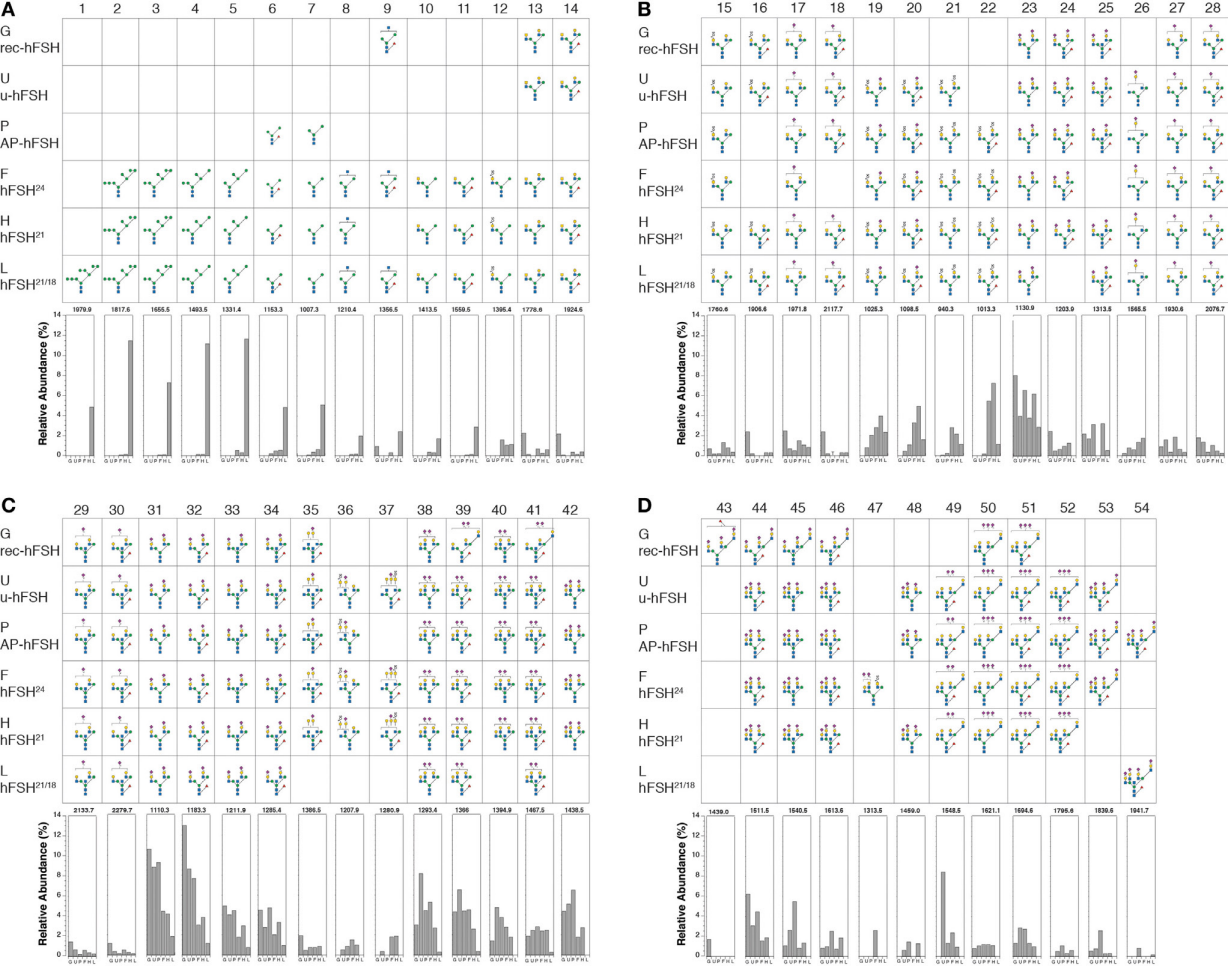
Human follicle-stimulating hormone (FSH) oligosaccharide microheterogeneity. Summary of results of nano-ESI mass spectrometry studies showing only those oligosaccharides present at >1% relative abundance in at least one hFSH preparation. The glycan diagram indicates it was detected in the preparation. The Consortium for Functional Glycomics monosaccharide symbols are used in conjunction with Oxford Glycobiology Institute linkage indicators (1–2, —; 1–3, \; 1–4, |; 1–6,/; solid lines indicate β-linkage and dashed lines indicate α-linkage). The bar graphs at the bottom of each panel indicate the relative abundance of the structure in each preparation. The preparations are indicated by single letters as follows: G is GH_3_-recombinant hFSH; U is urinary hFSH; P is pituitary hFSH; F is fully glycosylated pituitary hFSH^24^; H is hypo-glycosylated pituitary hFSH^21/18^; and L is hFSH^21/18^ isolated from hLH preparations. The structures are distributed across four panels beginning with the high mannose precursors and ending with tetra-antennary oligosaccharides, the largest found in hFSH. **(A)** Structures 1–14. **(B)** Structures 15–28. **(C)** Structures 29–42. **(D)** Structures 43–54.

The oligosaccharide structures shown in Figure 3 represent those present in at least 1% relative abundance in at least FSH preparation. Using this criterion, a total of 54 glycans were selected for comparison. The glycans are organized by position in the N-glycan biosynthetic pathway or by the number of complex branches. Within each antennary group, 2-, 3-, or 4-branch glycans, monosaccharide composition is the basis of organization. Structures 1–7 are oligomannose glycan intermediates found in ER and *cis*Golgi-derived glycoprotein precursors (Figure 3A). In multi-glycosylation site glycoproteins, these can be found in glycoproteins possessing mature glycans at other sites, when glycan processing at individual sites differs (30). Structures 8 and 9 exhibit the beginnings of complex oligosaccharide synthesis (Figure 3A), structures 10–34 are biantennary glycans (Figures 3A–C), structures 35–48 are triantennary glycans (Figures 3C,D), and structures 49–54 are tetra-antennary glycans (Figure 3D). The oligosaccharide populations of fully glycosylated FSH^24^ and hypo-glycosylated FSH^21^ preparations, F and D, respectively, possessed 51 of the 54 major glycans identified in these studies, and 45 of these, representing 88% of these more abundant glycans, were detected in both preparations. Pituitary and urinary FSH preparations P and U, respectively, both possessed 38 glycans (75%) in common with glycoforms F and D, while the hypo-glycosylated hFSH^21/18^ preparation L, possessed 35 glycans (68%) found in glycoform preparations F and D. Recombinant hFSH preparation G, expressed by stably transfected GH_3_ cells, displayed the lowest qualitative similarity to FSH^24^ and FSH^21^, possessing only 28 (55%) of the glycans found in glycoforms F and D. Moreover, the triantennary recombinant hFSH oligosaccharides displayed a different branching pattern.

Raising the cutoff to 4% relative abundance identified four groups of highly abundant glycans. The first group revealed a unique pattern of glycosylation for hFSH^21/18^ preparation L, consisting of a series of high mannose oligosaccharide intermediates possessing 9, 8, 7, 6, 5, and 3 mannose residues (structures 1–7, Figure 3A). Taken in isolation, this observation suggests that these glycoforms may not have exited the biosynthetic pathway. However, complex oligosaccharides, identical to those found in all other FSH preparations examined in this study, were also present in hFSH-L, suggesting oligosaccharide processing occurred at least at one glycosylation site in the Golgi. Glycosylation site-specific glycan analysis, when sufficient samples are available, or top–down proteomics for limited samples, have the potential to demonstrate the presence of both oligomannose and complex glycans in the same hypo-glycosylated hFSH molecule to support this hypothesis. Oligosaccharide structures 2–7 were also found in two pituitary glycoform preparations, hFSH^24^ and hFSH^21^. However, in both cases, these glycans were present in very low abundance, consistent with their being N-glycan biosynthetic intermediates. Moreover, both secreted hFSH preparations, urinary hFSH and recombinant hFSH, were devoid of oligomannose structures 1–7. In the case of urinary hFSH, this could have resulted either from rapid clearance of oligomannose-containing hFSH from the circulation or bias during purification.

As only secreted recombinant hFSH was recovered from conditioned medium, the absence of oligomannose glycans indicated that mature hFSH secreted by the GH_3_ cell line possessed only complex N-glycans. Moreover, the antibody used to capture recombinant hFSH appeared to capture all FSH forms, reducing the likelihood of purification biasing the oligosaccharide population (13). The high abundance of biosynthetic intermediate and low abundance of complex glycans in hFSH^21/18^ preparation L was notable because it exhibited the highest receptor binding-activity of any hFSH preparation we have studied. This led to the concern that we were studying a physiologically irrelevant glycoform. However, subsequent demonstration of significant biological activity differences between other pituitary and recombinant FSH glycoform preparations eliminated this concern (18, 31, 32).

Three clusters of high-abundance, complex glycans were noted in the other five hFSH preparations comprising oligosaccharide structures 22–23, 31–34, and 38–42. Group 2 structure 23, a disialylated, biantennary glycan possessing one GalNAc substituted for Gal, was highly abundant in all five preparations. This was notable, because the absence of sulfated GalNAc from hFSH N-glycans has been attributed to impaired recognition of a Pro-Leu-Arg motif in the common α-subunit of hFSH by β1,4-N-acetylgalactosaminyltransferase-T3 and -T4 (βGalNAct-T3 and βGalNAc-T4, respectively), as compared with hCG and hLH. The resulting reduction in FSH oligosaccharide sulfation was proposed as a consequence of altered motif access in this hormone, probably due to conformational change (33).

**Figure 4:**
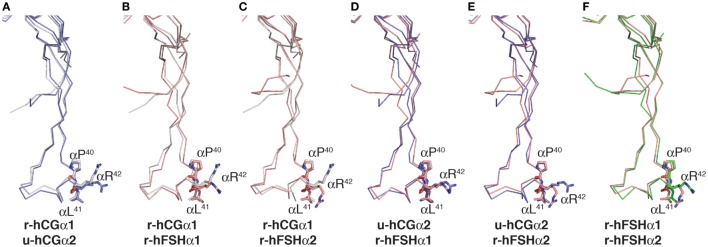
Comparison of Pro-Leu-Arg motif in hCG and follicle-stimulating hormone (FSH) crystal structures. Cystine knot loop α*L2* in the common α-subunits from each hormone structure were aligned using MacPyMOL. The backbone traces are shown and the side chains for Pro^40^, Leu^41^, and Arg^42^ shown as sticks. The residues are labeled because the flattening effect of printing appears to invert the order of Leu^41^ and Arg^42^. Chemically deglycosylated recombinant selenomethionine hCGα is r-hCGα1 (1hcn), chemically deglycosylated urinary hCGα is u-hCGα2 (1hrp), recombinant insect cell hFSH (1fl7) resulted in two models identified as r-hFSHα1 and r-hFSHα2, respectively. **(A–F)** α-subunit models aligned as indicated.

Comparison of Pro-Leu-Arg motifs in both hCG crystal structures, 1hcn (12) and 1hrp (11), with those in the two hFSH structures found in 1fl7 (6) showed positions of the Pro^40^ and Leu^41^ residue side chains were very similar in all six possible alignments (Figure 4). The Arg^42^ side chains were closely aligned in only one comparison, u-hCGα2:r-hFSHα1 (Figure 4D), suggesting flexibility in that region of the subunit (6, 34). Indeed, molecular dynamics simulations of FSH bound and unbound to the FSH receptor (FSHR) high-affinity binding site support flexibility in residue 40–47 region as unbound FSH exhibits root mean square fluctuations >1 Å (35). Unbound FSH is the form of the heterodimer recognized by β4GalNAc transferases. When FSH is bound to FSHR, this region loses flexibility, indicating it can achieve a stable conformation when bound to another protein. Thus, pituitary βGalNAc transferases are likely to bind this motif in both hLH and hFSH, consistent with the widespread distribution of GalNAc in hFSH oligosaccharides. The frequent appearance of GalNAc in sulfate-deficient glycans suggests an alternative hypothesis to explain reduced sulfation; human sialyltransferases compete more effectively with sulfotransferase in the human pituitary, leading to preferential addition of Neu5Ac to GalNAc. As N-glycan branches terminated with Neu5Ac-GalNAc were first reported for hLH oligosaccharides, finding this type of glycan is not unprecedented (36).

In fact, hLH possesses the greatest abundance of sialic acid of all characterized mammalian LH preparations (23, 36, 37). Moreover, structure 23 is part of a series of 15 GalNAc-containing, biantennary glycans observed in at least one of the six hFSH preparations (structures 10–25, Figures 3A,B). While two other structures are possible for the *m/z* 1130.9 ion associated with structure 23 (17), they do not permit addition of the two sialic acid residues associated with this oligosaccharide because the 5th hexosamine in the alternative structures is a bisecting GlcNAc residue and the single antenna possessing a Gal residue provides attachment for only one Neu5Ac residue. Group 3 glycan structures 31–34, are conventional, disialylated, biantennary oligosaccharides in which Neu5Ac residues are attached to Gal residues (Figure 3C). Structures 31 and 32 were the most abundant oligosaccharides derived from recombinant, urinary, and pituitary hFSH (Figure 3C). As 85–100% core-fucosylated glycans are found on the other human pituitary hormone LHβ and TSHβ subunits, structure 31 most likely reflects FSHα subunit glycosylation, while structure 32 reflects FSHβ subunit glycosylation (36, 38). The 4th high abundance glycan cluster, comprising structures 38–42, includes triantennary oligosaccharides possessing only two sialic acid residues. For this group of oligosaccharides, recombinant hFSH differed in the location of the two branch-mannose residues. In pituitary hFSH, GlcNAc transferase IV initiated a third glycan branch on Man (α1–3), while in recombinant hFSH GlcNAc transferase V initiated a third branch on Man (α1–6) (Figure 3C, compare row G with the other five rows). This suggested a difference in the relative activities of GlcNAc transferases IV and V between pituitary gonadotropes and somatotrope-derived GH_3_ cells, despite the expression of both transferase genes in GH_3_ cells (18). Another feature of recombinant hFSH glycans was antenna-linked fucose residues, such as observed in structure 43, one of the >1% abundance class of oligosaccharides (18).

## Impact of FSH Glycosylation Heterogeneity on Cognate Receptor Binding

**Figure 5:**
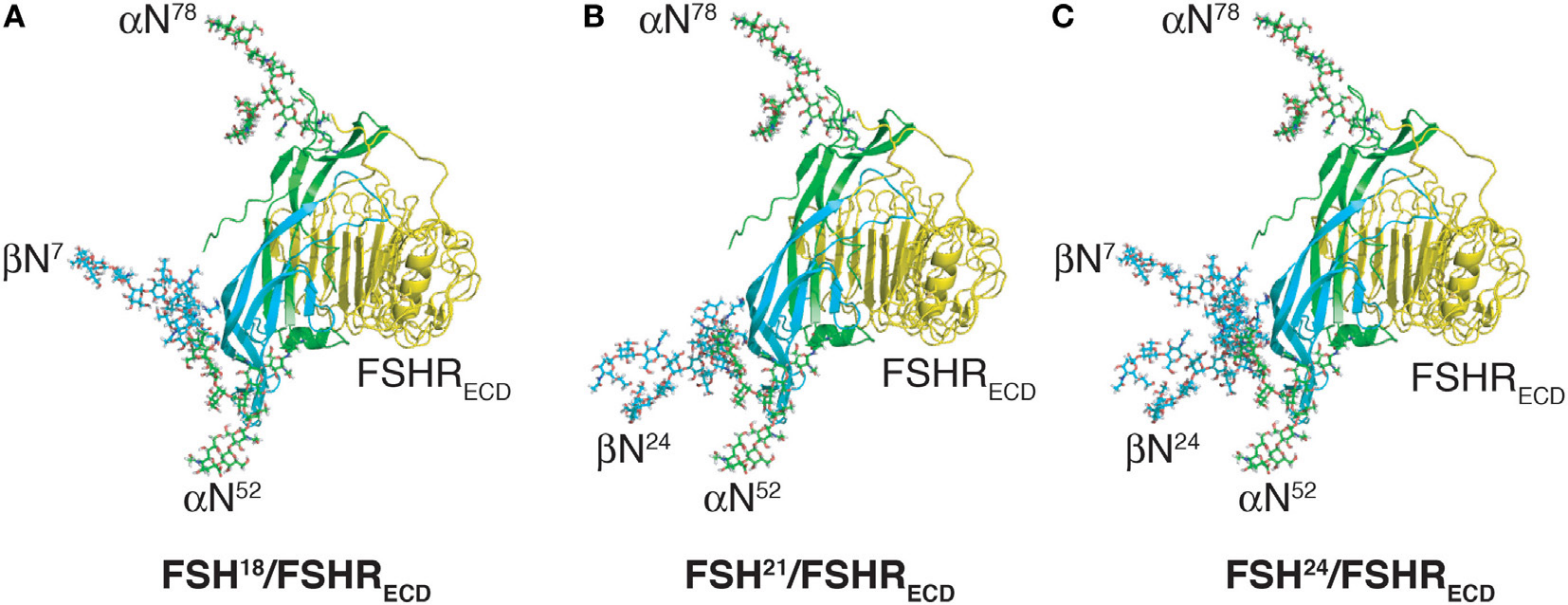
Follicle-stimulating hormone (FSH) glycoform models bound to monomeric FSH receptor (FSHR) extracelluar domain model (FSHR_ECD_). FSH glycoform models are oriented as in Figure 2. The FSHR_ECD_ model was extracted from pdb 4AY9 and rendered as cartoon using MacPyMOL. The FSH glycoform models were aligned to the FSH model extracted from the pdb file along with the FSHR_ECD_ to illustrate the positions of oligosaccharides relative to the high-affinity binding site in the FSHR. **(A)** Glycosylated model of FSH^18^ and FSHR extracellular domain. **(B)** Glycosylated model of FSH^21^ and FSHR extracellular domain. **(C)** Glycosylated model of FSH^24^ and FSHR extracellular domain.

The FSHR is a G-protein-coupled receptor (GPCR) with a leucine-rich repeat extracellular domain comprising 358 amino-acid residues. This ligand binding domain is connected to a 337-residue, hepta-helical transmembrane domain (39, 40). Crystal structures of the high-affinity FSH binding domain in complex with FSH revealed that the interface of the complex involves contacts exclusively *via* protein–protein interactions (41, 42). FSH oligosaccharides added by modeling do not appear to interact with the extracellular domain engaged with FSH, as they are located on a face of the hormone, which is oriented away from the hormone receptor interface (Figure 5). Since it is well established that FSH carbohydrate is necessary for full FSHR activation (43–46), it seems reasonable to assume that the carbohydrate affects hormone conformation, which in turn modulates activity. The structure of the entire FSHR (extracellular domain and transmembrane domains) in complex with FSH has yet to be determined, and until then, carbohydrate interaction with the transmembrane domain cannot be ruled out. Alternatively, carbohydrate modulation of FSH conformation may affect the final disposition of FSHR extracellular domain (FSHR_ECD_) hinge region putative interactions with extracellular loops of the transmembrane domains (34, 47). Consistent with the absence of FSH carbohydrate interaction with FSHR_ECD_, isolated hybrid-type oligosaccharides related to structure 12 in Figure 3 have no effect on FSHR binding (48). Nevertheless, these oligosaccharides significantly inhibit both basal granulosa cell steroidogenesis, as well as FSH-stimulated steroidogenesis (48). The low affinity of carbohydrate–protein interactions requires sufficiently high oligosaccharide concentrations in inhibition studies that hormone contamination can inhibit binding assays. In our hands, a minimum of two purification steps is necessary to eliminate residual hormone assay interference (48). Accordingly, we attributed hormone contamination in the oligosaccharide preparation as the reason for a report that hCG-derived oligosaccharides inhibited both receptor binding and cellular activation (49).

Loss of a single FSHβ oligosaccharide has three effects on FSH binding to its receptor. First, hypo-glycosylated hFSH immediately engages FSHR preparations, whereas fully glycosylated hFSH^24^ exhibits about a 30-min lag before FSHR binding begins in earnest (13). Second, hypo-glycosylated hFSH^21/18^ exhibits a 2.8- to over 14-fold higher apparent affinity for the FSHR as compared with hFSH^24^ (Table 2). Third, hypo-glycosylated hFSH^21/18^ occupies 2- to threefold more FSHRs than FSH^24^ (13, 18). A glance at the structures of FSH glycoforms bound to the FSHR_ECD_ immediately raises the question of how loss of either FSHβ N-glycan facilitates FSH association with the receptor, as neither glycan is close to the binding site (Figure 5). This leaves yet to be defined hindrance by the FSHR transmembrane domain or FSHR oligomerization as potential mechanisms.

**Table 2:** Follicle-stimulating hormone (FSH) receptor-binding activities of pituitary and recombinant hFSH glycoform preparations.

FSH preparation	Pituitary hFSH	Urinary hFSH	Pituitary hFSH^24^	Pituitary hFSH^21/18^	Recombinant GH_3_ hFSH^24^	Recombinant GH_3_ hFSH^21^
FSH RLA potency (IU/mg)	8,560	10,000	18,737	269,445	20,844	57,942

FSH^21^/FSH^24^ ratio			14.4		2.8	

*The radioiodinated tracer was 2.5 ng/tube ^125^I-hFSH and the receptor preparation was 250,000 FSHR-expressing CHO cells/tube*.

*Data derived from Ref. (16, 18)*.

The crystal structure of the high-affinity binding site of the FSHR_ECD_ comprised two FSHR domains associated back to back, sandwiched by FSH ligands (41). There was no indication of FSH oligosaccharide interaction with the receptor. The crystal structure of the entire FSHR_ECD_ with FSH bound revealed a strikingly different FSHR_ECD_ conformation as trimeric FSHR–FSH complexes (42). To obtain diffractable crystals in both studies, endoglycosidase-F digestion reduced FSH and FSHR_ECD_ N-glycans to single GlcNAc residues, which eliminated oligosaccharide influence on hormone-receptor binding. The trimeric FSHR crystal structure suggested FSH αAsn^52^ oligosaccharide, when present, would restrict ligand binding to one glycosylated FSH ligand per FSHR trimer as a biantennary glycan attached to this Asn residue would occupy the center of the trimeric complex (47). While no subsequent studies supporting the dimeric FSHR model have been reported, several lines of evidence appear to support the trimeric FSHR_ECD_ model. Biochemical data in support of the trimeric FSHR model were provided when recombinant-mutant des-αN^52^-hFSH exhibited threefold greater binding to CHO cells expressing hFSHRs as compared with recombinant wt-hFSH (47). Small molecule allosteric FSHR modulators were reported to increase FSH binding ~threefold, suggesting trimeric FSHR complexes dissociating to form FSHR monomers (50–52). Incorporating a transmembrane domain model to the FSHR_ECD_ trimer model predicted that only a single β-arrestin could bind to the trimeric FSHR. Addition of an allosteric modulator to β-arrestin binding assays produced a threefold increase in β-arrestin binding, supporting a model that allosteric small molecule FSHR modulators dissociate FSHR trimers into monomers, thereby increasing FSH access (47). However, a superresolution microscopic technique, dual-color photoactivatable dyes, and localization microscopy (PD-PALM) revealed the closely related LHR existed as a variety of oligomeric forms as well as monomers in the cell membrane (53). Docking of complete LHR models in this study provided a variety of conformations of LHR oligomers, including trimeric LHRs. Similar studies with FSHRs would help clarify the relationship of FSHRs.

As greater FSHR occupancy is directly proportional to FSH-stimulated cAMP production by target cells, increased hypo-glycosylated hFSH binding to FSHR is expected to provide a correspondingly greater cellular activation than fully glycosylated hFSH (54). However, since the model of an FSHR trimer can only accommodate one G protein, it is unlikely that the increase in cAMP is due to occupancy alone. Another possibility is that occupancy by hypo-glycosylated FSH fails to engage the GRK/arrestin pathway which would otherwise attenuate the reengagement of G protein subsequent to activation of adenyl cyclase. Another possibility is that hypo-glycosylated FSH creates a more stable complex with FSHR such that during intracellular trafficking, cAMP- and arrestin-mediated persistent signaling (55) is enhanced. Finally, one may also suggest that since the FSH/FSHR complex appears to recycle to the cell surface (56, 57), the high-affinity binding of hypo-glycosylated FSH may have a proclivity for FSHR, thus failing to dissociate upon relocation to the plasma membrane and perhaps reformation of the putative trimeric structures. This could affect the dynamic stoichiometry of the cell surface unoccupied receptor cohort whose ontogeny resets not only with new FSHR synthesis but also by occupancy/recycling engaged by other members of the orchestra^1^ of glycoforms.

## Fshr-Mediated Signaling *in vitro* and *in vivo*

Biased signaling has underpinned GPCR drug development for years but only recently has the mechanism of this phenomenon been revealed in the GPCR field, including the FSHR (51, 58–60). The realization that one GPCR can activate several effector proteins to activate different pathways has prompted the challenging of previously accepted dogma and may help to explain previously unexplained observations. An example of such dogma is that both FSHR and LH/CGR primarily signal *via* Gαs leading to the activation of the cAMP/protein kinase A (PKA) pathway and subsequently leading to steroidogenesis (51, 61–64). Alternative pathways, such as phospholipase C/inositol trisphosphate metabolism were first recognized over 25 years ago (65, 66); however, most studies examining the actions of gonadotropin glycosylation variants remain fixed on the primary pathway. The concept of biased signaling predicts that the specificity of signal transduction depends on, at least in part, the structure of the ligand [reviewed in Ref. (58, 59)]. In support of this idea, a partially deglycosylated eLH variant (67) (eLHdg) was found to exhibit biased signaling through the FSHR (68). While incapable of activating the cAMP/PKA pathway and eliciting steroidogenesis in granulosa cells, binding of eLHdg to FSHR recruited β-arrestins and activated ERK MAPK signaling *via* a cAMP-independent pathway (68).

Another recent study showed that the oligosaccharide complexity of recombinant hFSH preparations differentially affected gene expression and steroidogenesis in human granulosa cells (69). Our own studies with hFSH glycoforms have found evidence for biased signaling, albeit in different cell types. The hFSH^21/18^ glycoforms were more active than hFSH^24^ in activating the cAMP/PKA pathway and phosphorylation of PKA substrates *via* Gαs in human KGN granulosa cells (31). The actions of FSH^21/18^ were 10-fold greater than FSH^24^ on induction of CYP19A1 and estrogen (31). The obvious next step is to determine if this biased signaling by hFSH^24^ occurs in gonadal cells, which is an active area of pursuit using both *in vitro* and *in vivo* genetic approaches.

## Genetic Models to Study the Physiology of FSH Glycoforms

### *Fshb* Knockout Mice

As mentioned above, hypo-glycosylated FSH^21/18^ has been shown to be more avid compared with fully glycosylated FSH^24^ in several receptor binding assays (13, 18), and more potent when tested using primary granulosa cell- or immortalized granulosa cell-based *in vitro* assays (31). Translation of these *in vitro* observations from biochemistry to physiology required the development of new models as well as implementation of existing mouse models. Accordingly, *in vivo* effects of FSH glycoforms FSH^21/18^ and FSH^24^ were evaluated using the experimental design of an *in vivo* pharmacological rescue approach. In this experimental paradigm, first, immature *Fshb* null female mice (at 21 days of age) were injected i.p. with different doses of FSH glycoforms separately and at different times postinjection, ovaries were collected for subsequent selected gene expression analysis by quantitative real-time PCR. In these studies, hypo-glycosylated FSH^21/18^ elicited *in vivo* bioactivity comparable to that of FSH^24^; however, these analyses also indicated that differences exist between FSH^21/18^ and FSH^24^ glycoforms in inducing a unique subset of FSH-responsive genes (32). Second, to assess the upstream signaling pathways which control FSH-induced gene expression, immunofluorescence analysis was performed on ovarian sections obtained from *Fshb* null female mice injected with FSH^21/18^ and FSH^24^ glycoforms using p-CREB and p-PKA substrate antibodies. At three different time points tested (0.5, 1, and 2 h), both glycoforms were equally effective and significantly upregulated p-PKA and p-PKA substrates (nuclear accumulation in granulosa cells) over PBS-injected controls, with maximal induction observed at the 1-h time point (32).

In a third set of experiments, ovarian protein extracts were obtained from *Fshb* null female mice at different time points after injecting with FSH glycoforms separately. These extracts were subjected to Western blot analysis followed by densitometry quantification. When induction of p-CREB, p-PKA substrate and p-p38, p-p44/42, and p-AKT was compared, FSH^21/18^ hypo-glycosylated FSH, similar to the above assays, was as active as that of FSH^24^, the fully glycosylated FSH (32). Finally, in ovarian weight gain response assays, FSH^21/18^ was equally potent as that of the FSH^24^, although FSH^21/18^ elicited better estradiol induction compared with that by FSH^24^ (32). Thus, the *in vivo* pharmacological rescue experiments suggest biased agonism exhibited by different FSH glycoforms and, as would be expected, these are nuanced. In addition to determining if this phenomenon occurs *in vivo* as a function of age (particularly in regard to bone density given the correlation of age with changing FSH glycoform abundance), it will also be critical to determine if these nuances correlate with fertility or embryo quality, having great potential impact on therapeutic use.

*In vivo* pharmacological rescue of *Fshb* null male mice was also performed using recombinant human FSH glycoforms and measurement of testicular weight gain between postnatal day 5 and 10 in *Fshb* null male mice (32). When injected separately into *Fshb* null male mice at postnatal day 5, both FSH glycoforms significantly induced testicular weight gain by day 10 compared with that in PBS-injected controls (32). Testis weight correlated well with testis tubule size, as well as number of germ cells per tubule. Hypo-glycosylated FSH^21/18^ was more active than FSH^24^ (32). Similarly, a subset of FSH-responsive genes in mouse Sertoli cells responded much better to hypo-glycosylated FSH^21/18^ than fully glycosylated FSH^24^. Furthermore, the number of BrdU^+^ Sox9^+^ proliferating Sertoli cells was also found significantly higher in testes of mice injected with FSH^21/18^ compared with FSH^24^ (32). It is likely that different human FSH glycoforms act *via* different FSHR-mediated downstream signaling pathways in mouse Sertoli cells, similar to granulosa cells, and elicit distinct gene/protein expression changes. These observations suggest there may be a therapeutic potential advantage of using glycoform-specific hFSH preparations for treatment of male factor fertility, such as marginal sperm counts.

### Evaluation of FSH^15^ in *Fshb* Null Mice

*In vitro* expression, purification and characterization of recombinant human FSH glycoforms in somatotrope-derived GH_3_ cells often results in FSH^21/18^ and FSH^24^ as the most abundant FSH glycoforms identified by mass spectrometry (18). However, according to the all or none FSHβ glycosylation concept, FSH dimers containing non-glycosylated FSHβ (expected to be 15 kDa in denaturing gels) could also exist in pituitaries (20). To test the biological significance of non-glycosylated FSHβ, separate lines of transgenic mice were first generated that expressed, either a human *FSHB*-mutant transgene (*HFSHB*^7Δ24Δ^) encoding a glycosylation defective 15k-FSHβ subunit or a human *FSHB* WT transgene (*HFSHB^WT^*)-encoding wild-type (WT) FSHβ subunit, specifically in gonadotropes. The transgenes were subsequently introduced onto an *Fshb* null genetic background by intercrossing using a genetic rescue strategy (70).

Real-time qPCR assays, immuno co-localization, and Western blot analyses under denaturating conditions confirmed that the transgene encoded mRNA and the corresponding subunits were abundantly expressed in pituitaries (21). While WT human FSHβ subunit-containing, inter-species hybrid FSH was readily detectable by Western blot analysis under non-denaturing conditions of *HFSHB ^WT^* mouse pituitaries, FSH dimer containing double N-glycosylation-mutant human FSHβ subunit was barely detectable in pituitaries of *HFSHB^WT^* mice on an *Fshb* null genetic background (21). Consistent with these expression data, mutant FSHβ subunit-containing FSH dimer was not detectable in either short-term pituitary organ culture media or serum samples by specific RIAs (21). Furthermore, gonad histology, gonad gene expression, and fertility assays all indicated that the double N-glycosylation-mutant *HFSHB* transgene failed to rescue *Fshb* null mice (21). Taken together, these genetic experiments confirmed that the double N-glycosylation-mutant human FSHβ subunit-containing FSH dimer is unstable *in vivo*. Such a dimer is also secretion incompetent and even when secreted in low amounts, it fails to rescue mice lacking FSH. Thus, at least one N-glycosylation site on human FSHβ subunit is essential for efficient FSH dimer assembly, secretion, and biological activity *in vivo*.

## Summary of Integrated Results

### Implementation of Glycoforms in ART/IVF

Fundamental and heretofore unrecognized differences in human FSH relating to the number and location of FSH glycans resulting in FSH glycoforms, FSH^24^, FSH^21^, and FSH^18^ (16, 19) have been summarized. Moreover, the seminal observation from analysis of individual human pituitaries was that the abundance of FSH^21^ declines with age in women [Table 3 and (16)] raises the question whether this had implications for therapeutic intervention. FSH^21^ is elevated in young women of reproductive age, but declines thereafter leading to a condition of FSH^24^ dominance. Thus, the active reproductive period is characterized by the presence of FSH^21^, while the period of declining fertility and reproductive senescence is characterized by significantly diminished FSH^21^ along with FSH^24^ dominance.

**Table 3:** Relative abundance of FSH^21^ in individual human pituitaries.

No. of pituitaries	2	4	4
Age range (years)	21–24	39–43	58–71
FSH^21^	62 ± 10.5	41 ± 8.2	17 ± 3.7

*Based on band density in Western blots using anti-FSHβ monoclonal antibody RFSH20 (16)*.

In this regard, it is noteworthy that current hFSH products available commercially for clinical use, whether they are of menopausal or recombinant origin, consist overwhelmingly of FSH^24^ (18). Thus, despite the general success of IVF, there has not been a systematic clinical trial which considers that a form of the hormone associated physiologically with a period of decreased reproductive function rather than the form of the hormone present during the reproductive period may be compromising both yield and quality of embryos. It is believed that the clinical utilization of hypo-glycosylated FSH^21/18^ preparations for IVF would represent a paradigm shift in the treatment of infertility. The use of something truly different, an apparently more active and more physiologically relevant FSH, might provide the basis for improved ovarian stimulation and overall pregnancy outcome. Thus, an emerging question is whether the shift from FSH^21^ to FSH^24^ dominance occurs as a result of normal aging or a premature change and represents an underlying cause of subfertility/infertility. To place this in context, a brief overview of controlled ovarian stimulation (COS) is warranted.

The use of COS began in the 1980s as a means to enhance/improve the chances of generating a pregnancy *via* the combination of procedures involved in *in vitro* fertilization (IVF). Prior to this, “natural cycle” IVF was utilized, which generated on average, a single utilizable oocyte (71). Not surprisingly, success *via* this method was severely limited. COS was developed as a means to generate multiple oocytes, which would increase the chances for successful fertilization, enhance embryo development, and coupled with multiple embryo transfer to the uterus, increase pregnancy rates. Indeed, COS proved invaluable as the preferred mechanism underlying IVF (72, 73). In parallel to COS, increased focus on IVF Laboratory practice coupled with IVF Laboratory Certification greatly moved IVF from “experimental procedure” status to that of standard of care (74). At the core of COS is the utilization of hFSH, the fundamental endocrine driver of ovarian follicle development (72).

The history of COS has witnessed a number of modifications aimed at increasing IVF success. Among these are: the utilization of GnRH agonist or antagonists to block endogenous gonadotropin production; utilization of urinary-derived human menopausal gonadotropin or well-controlled recombinant cDNA-driven expression of hFSH produced primarily using cells of Chinese hamster ovary origin; the use of FSH alone or the combined use of FSH coupled with LH; variable gonadotropin dosage and administration regimens; and utilization of supplemental progesterone to offset or oppose estradiol levels (72, 73, 75). Often, modifications have been undertaken to treat women with special conditions that impact success including women with PCOS, older women, and women with cancer (76). Indeed, a women’s age is one of the most predictive factors underlying success with IVF due in large part to the diminishing pool of primordial follicles. The common and overriding feature of the above modifications is the utilization of FSH.

The mechanistic functions and potential differences among FSH glycoforms remain largely unknown. As noted above, differences in receptor binding and the subsequent impact upon certain intracellular signaling systems and cell function can and have been demonstrated (77, 78). The fundamental mechanisms underlying female fertility in terms of producing a viable oocyte still remain largely unknown. However, there are clearly defined stages, which offer targets for differential regulation. These stages include primordial follicle activation, preantral follicle growth, antral follicle growth, and dominant follicle selection. An intriguing hypothesis is that hFSH glycoforms function during different stages of follicle development. This might explain in part, the reported differences in glycoform stimulation of ovarian gene expression and cellular signaling pathways observed in the immature *Fshb* null mice (32).

Follicle development up to the antral stage is not dependent upon FSH in the mouse (79, 80). Nevertheless, preantral follicles are responsive to FSH (81, 82). Owing to the recently reported *in vivo* activities of the glycoforms, could FSH^21/18^ preparations function to drive preantral follicle development to provide follicles appropriately responsive to FSH^24^? Might supplementation with FSH^21/18^ for one or two cycles prior to COS overcome what appears to be a natural decline in fertility with age concomitant with a decline in the levels of hFSH^21/18^? One proposes supplementation in the event that FSH^21/18^ drives preantral follicle development, so that replacement of FSH^24^ by FSH^21/18^ under standard COS strategies may not provide for improved results if FSH^21/18^ is needed during the earlier stages of follicle development and ineffective in later stages. Furthermore, such treatment paradigms might serve to ameliorate the decreased responsiveness of older women to COS with commercially available FSH, which is essentially FSH^24^. There is, for example, some evidence that microheterogeneity differences affect estradiol production (78).

FSH^21/18^ supplementation over an extended period to promote preantral follicle development, which would serve to provide appropriately developed follicles for continued development, perhaps with either glycoform. Owing to potential differences in uptake and circulating half-life, and whether the glycoforms are under episodic as opposed to a more tonic secretion, differences in hFSH glycoform dose and administration regimen may be needed to provide for a more physiological representation. Clearly, the discovery of FSH^21/18^ and the initial characterization of its activity provide the basis for new ideas concerning COS and IVF. These data indicate that FSH^21/18^ and FSH^24^ exist, and they exhibit differences in both *in vitro* and *in vivo* activities, and their relative abundance changes with age. These data provide a compelling basis for continued investigation. Central to the improvement of IVF outcomes will be the understanding of how and when these two glycoforms function to promote the proper developmental program of the follicle.

### Implementation of FSH Glycoforms to Preserve Bone

Follicle-stimulating hormone has been reported to have direct effects on bone, attributed to FSH-driven (83–85) osteoclast development and activity (86–89). During the premenopausal period, when ovarian reserve is waning and FSH levels are rising because of the lack of negative feedback by ovarian estrogen (90), the abundance of fully glycosylated hFSH^24^ in the pituitary also rises. It is well established that declining levels of estradiol during the menopausal transition affects bone mineral density, and other metabolic parameters (91). Since the 1940s it has been assumed that reduced bone mineral density was due to a simple sex steroid deficiency (92). Previous reports, largely from one laboratory, have challenged this view by providing evidence that elevated FSH during menopause or ovarian deficiency might explain the bone loss (86, 93). A number of observations highlight the potential importance of FSH in mediating, at least in part, bone loss in humans (94) not associated with changes in steroid hormones (84). A recent study found that FSH, but not estrogen, was strongly associated with bone loss in postmenopausal women treated for breast cancer (95). Furthermore, polymorphisms in the *FSHR* are associated with accelerated bone loss in women (96). As such, the levels of estrogen and FSH may contribute in multiple ways to bone mineral density during aging.

It should be appreciated that the extra-gonadal actions of FSH have only been recently identified and the actions of FSH on bone have been controversial [reviewed in Ref. (97)]. Allan et al. (98) reported that FSH produced anabolic effects on bone that correlated with inhibin and testosterone levels. Ritter et al. (99) found that treatment of mice with FSH had no effect on bone loss or gain and did not increase osteoclast formation. Two other groups found little correlation of FSH levels and bone mineral density (2, 100). In contrast, other studies provide evidence that FSH can promote the development of human osteoclast precursor cells (89) and induce the production of bone-resorbing cytokines (87, 88, 93). These are relevant observations since the immune system plays a role in a variety of disease states linking inflammatory responses and bone loss (101). Furthermore, several lines of evidence support the initial observations that loss of either *Fshb* or *Fshr* confers protection from bone loss in mice (86).

Geng et al. (102) showed that exogenous FSH enhanced osteoclast differentiation and treatment with neutralizing antibodies to FSH or a GST–FSHβ fusion protein prevented bone loss in ovariectomized rats. Likewise, Zhu et al. (103) reported that treatment of ovariectomized mice with an FSH antibody prevented bone loss. Our data show that treatment of murine and human osteoclast precursor cells with FSH^24^, but not FSH^21^, increases the formation of multi-nucleated, TRAP (tartrate-resistant acid phosphatase-5b, a bone resorption marker) positive osteoclasts (Davis et al., unpublished). FSH also works together with receptor activator of nuclear factor-κB (NFκB) ligand (RANKL) to induce expression of MMP9 and cathepsin-k (CTSK) in osteoclasts. These data are in agreement with our own and indicate that FSH^24^ increases *TNF*α *and IRAK* mRNA in human CD14^+^ osteoclast precursors. TNFα is important for osteoclast formation (93, 104, 105). These findings indicate that the age-related increase in hFSH^24^ may regulate bone, a nontraditional FSH target. Evidence points to the ability of FSH to activate Gα_i_ in bone cells, resulting in a reduction in cAMP levels (86), which contrasts to the activation of Gα_s_ and increase in cAMP in granulosa cells. In bone, FSH stimulates MAPK and NFκB osteoclastogenic intracellular signaling pathways (86). Our data indicate that FSH^24^ is responsible for activating these signaling pathways and formation of osteoclasts. Hence, there is a critical need to settle the controversy regarding a role for FSH in targeting osteoclasts in women.

## Author Contributions

GB, JM, JSD, JD, and TK authored individual sections of the review. GB, JM, JD, JD, and TK reviewed the entire manuscript.

## Conflict of Interest Statement

The authors declare that the research was conducted in the absence of any commercial or financial relationships that could be construed as a potential conflict of interest.

## Footnotes

^1^Each of the FSH molecules is analogous to a member of the orchestra. Their seat is the receptor and their glycoforms are the instruments which they bring to play. The role of each molecule in the orchestra performance will be dependent on the glycoform instrument they bring with them. Like an orchestral performance, one must envision FSH signaling as a complex symphony which may be deconstructed but with loss to the nuance and impact of the full symphony.
